# Construction and application of economic resilience evaluation model for megacities

**DOI:** 10.1371/journal.pone.0301840

**Published:** 2024-05-24

**Authors:** Chenhuan Kou, Donghan Meng, Xiuli Yang

**Affiliations:** 1 School of Management Science and Engineering, Hebei University of Economics and Business, Shijiazhuang, Hebei Province, China; 2 Development Planning Department, Hebei University of Economics and Business, Shijiazhuang, Hebei Province, China; 3 School of Management science and Engineering, Central University of Finance and Economics, Beijing, China; 4 School of Public Administration & Law, Northeast Agricultural University, Harbin, Heilongjiang, China; Sichuan Agricultural University, CHINA

## Abstract

Economic resilience provides a new perspective for megacities to achieve sustainable development when facing multiple shocks, and its accurate evaluation is an essential prerequisite for optimizing urban governance. There are currently no generally accepted methods for empirical evaluation or measuring economic resilience, and the present study aims to contribute to in both the research field and methodology. The present study sets dimensions and indicators based on economic resilience’s theoretical and empirical research and used Decision Making Trial and Evaluation Laboratory (DEMATEL) and Interactive Structural Modeling (ISM) methods to exclude the effect indicators and divide the indicator hierarchy, respectively. Subsequently, the present study conducts model validation using Chinese megacities as a case study. The game theory weighting method, which combines the Analytic Hierarchy Process (AHP) and Entropy methods, is used to calculate indicator weights, and the VIKOR (VIseKriterijumska Optimizacija i KOmpromisno Resenje) method is used to evaluate and compare economic resilience of megacities. The research findings indicate that the evaluation model constructed in the present study included 15 indicators (after excluding three effect indicators) divided into four levels. After merging the levels, they correspond to three dimensions: resistance, recoverability, and adaptability. In addition, using Chinese megacities as a case study, the evaluation results found that Beijing, Shanghai, and Shenzhen have high economic resilience, Tianjin and Guangzhou have moderate economic resilience, Chengdu has low economic resilience, and Chongqing has the lowest economic resilience. This result is consistent with previous studies and verifies the model’s effectiveness. The present study also found that megacities with lower levels of economic resilience exhibit a more significant upward trend, as well as the highest and higher proportion of economic resilience in Chinese megacities depending on time passes, indicating that megacities’ economic resilience is weakening. The evaluation result obtained in the present study is more specific, precise, and focused on depicting the distribution differences and development trends of economic resilience at the urban level.

## Section 1: Introduction

Recently, cities generally have faced increasing problems such as global warming, environmental pollution, earthquakes, floods, and disease [1, 2]. As the economic, political, innovation, and cultural centers of region and nation, megacities gather a large amount of resources and population while increasing their complexity and vulnerability [3, 4]. Risks and shocks, once they occur, will quickly spread along the urban network, resulting in cascading failures. For example, COVID-19 broke out at the end of 2019 and originated in the health sector but then quickly spread to the entire socio-economic network, causing the global economic recession since the 2008 financial crisis [5].

Economic resilience is a significant performance and a key indicator of sustainable economic development of megacities [6–9]. It describes whether or not an economic entity can quickly adapt and recover from various shocks [10–13]. Economic resilience encompasses at least three abilities of an economic entity: resistance, recoverability, and adaptability [14]. Thus, megacities with economic resilience are not only able to resist crisis and quickly recover to the pre-crisis growth path but also even being able to adjust economic structure and explore more long-term economic growth models through crisis as opportunities.

Accurately evaluation is an essential prerequisite for relevant policy formulation and governance optimization, but there are no generally accepted methods for empirically evaluating or measuring economic resilience [15]. The two commonly used quantification methods are the single-factor measurement method and the comprehensive indicator method [16]. The single-factor measurement method reflects the strength of economic resilience by calculating change intensity in a specific factor, usually selecting employment rate [4, 17, 18], unemployment rate [19], GDP [20, 21], and foreign trade [22]. For example, Martin (2012) measured and compared the economic resilience of different regions in the UK by calculating the change degree of employee rate [12]. In contrast, the comprehensive indicator method constructs an index system to evaluate economic resilience. For example, Briguglio et al. (2006) built an index system from dimensions of macroeconomic stability, microsubject efficiency, economic system rationality, and social development stability, which is applied to evaluate 80 countries’ economic resilience [23]. Since the comprehensive indicator method considers the marginal contribution of more factors, it is widely used by various organizations, such as the Centre for Local Economic Strategies (CLES) and ARUP, compensating for the shortcomings of the single-factor measure method to a certain extent.

The comprehensive indicator method still has some gaps that need to be filled. The indicator selection needs to be more rigorous, effect indicators need to be excluded, and the indicators’ hierarchy needs to be classified. With this in mind, the present study aims to fill these gaps and contribute to the research scale and methodology. Firstly, the present study further expands the research scope of economic resilience from regional to urban areas, focusing on the economic resilience issues of megacities. Secondly, the present study further optimized the comprehensive indicator method to enhance its effectiveness and strictness. Specifically, the present study sets evaluation dimensions and indicators based on theoretical and empirical research on economic resilience. Then, the present study comprehensively uses DEMATEL and ISM methods to exclude effect indicators and divide the indicators’ hierarchy. Afterward, the present study takes China’s megacities as examples to verify the model’s effectiveness, and the game theory weighing method that combines AHP and Entropy methods is adopted to calculate indicator weights, and the VIKOR method is used to evaluate and compare the economic resilience of Chinese megacities.

The remaining of the present study is organized as follows. The next section introduces the cognitive evolution, definition, and mainstream evaluation methods of economic resilience. Section 3 elaborates on indicator selection, indicators’ causality identification, indicators’ hierarchy division, and the evaluation model. Section 4 takes Chinese megacities as a case study and combines indicator weighting and fuzzy decision-making to verify the model. Section 5 presents a brief discussion to analyze the results difference between the present study and previous studies. The last section concludes the research findings, proposes policy implications, and points out the research limitation of the present study.

## Section 2: Review of the literature

The word “resilience” originated from the Latin word “resilio”, which means “to bounce back.” By the 16th century, the French borrowed the word “resilio” and produced “résiler,” which was used to indicate rebound or withdrawal, and it gradually evolved into “resilience” in modern English. The concept of resilience has experienced three extensions, from engineering resilience and ecological resilience to evolutionary resilience (adaptive resilience) [24, 25]. Economic resilience is the application of resilience thinking in the field of economics. The following context reviews relevant research on economic resilience concepts and evaluation methods.

### Economic resilience concept

With the concept expansion of resilience from engineering resilience to ecological resilience to evolutionary resilience, two mainstream perspectives have emerged for interpretating economic resilience: the equilibrium-based perspective and the evolutionary perspective. The following is a specific analysis:

#### Economic resilience from an equilibrium-based perspective

Economic resilience from an equilibrium-based perspective comes from engineering and ecological resilience. Engineering resilience considers that there is only one stable state in the system, and resilience refers to the ability of the system to maintain a steady state after being disturbed or returning to a stable condition after deviation. Ecological resilience believes that there are multiple problems in the system [26], and the system may return to the initial steady state and cross the threshold to reach another stable state after an external disturbance. Therefore, economic resilience in this scenario refers to the ability of an economic system to maintain normal functions or gradually recover to the original development path when facing shocks [27].

Economic resilience originated from engineering resilience, which believes the economic system has one ideal development state. Shocks will stimulate the system’s ability to self-adjust and recover to the perfect state. In other words, a system with high economic resilience can resist shocks and self-recover, maintaining a balanced development trajectory.

Economic resilience originated from ecological resilience, which believes the economic system is a complex self-organized system with multiple equilibrium states. If the shock intensity exceeds the maximum magnitude the economic system can withstand, the system will undergo a steady transition and change from one equilibrium state to another. Therefore, the system with high economic resilience can switch to another development path under the influence of shocks, but the system with low economic resilience lacks this ability.

#### Economic resilience from an evolutionary perspective

Economic resilience from an evolutionary perspective comes from evolutionary resilience. With further study of the system, scholars have realized that the social-ecological system constantly changes and is more similar to a complex adaptive system and may not have a steady state. Therefore, compared with engineering and ecological resilience, evolutionary resilience is a new kind of cognition that has broken the long-term pursuit of balance and stability in resilience research and emphasizes the system’s adaptive learning, innovation, and transformation capabilities [28, 29].

Economic resilience originated from evolutionary resilience and is recognized as an inherent attribute of the economic system that changes dynamically. When facing shocks or disturbances, the system with high economic resilience can maintain normal functions, recover rapidly, and adjust system structure and institutional arrangement to achieve long-term growth, if necessary.

Specifically, economic geographers think that economic resilience is embedded in the dynamic evolution process of the economic system. It is not formed when shocks occur but is a historical path dependence constantly developed and strengthened under the alternating influence of the external environment and historical heritage. Economic resilience covers the economic structure, production relations, institutional arrangements, innovative culture, etc. [11, 15]. It is difficult to adjust or change once the regional historical path dependency is finalized. As Weitzman described in the Recombinant Growth Theory that was put forward in the 1990s, it is challenging to cultivate new industries in a region out of thin air. They must be born based on resource accumulation, knowledge endowment, and custom culture formed through long-term development in the area [14]. From this point of view, economic resilience from the perspective of evolutionary theory is closely related to economic and innovative geography.

To summarize, the economic system is an open and complex adaptive system, showing the characteristics of non-linearity, adaptability, dynamics, development, and self-organization. The individual or multiple steady states assumed by economic resilience from an equilibrium-based perspective have certain limitations, making explaining economic resilience’s diversity and uneven distribution challenging. From the standpoint of evolutionary theory, economic resilience deeply analyzes the economic system’s dynamic process and historical path dependence, and it has become a research hotspot in the current academic circle. Previous studies have conducted an in-depth analysis of the characteristics and connotations of economic resilience using various research methods, such as literature research, case studies, econometric models, network analysis, etc. These studies contribute to revealing the response mechanism of economic resilience to shocks by studying the knowledge endowment and cultural context accumulated in the long-term historical evolution process, which is used to identify the reasons behind the economic system’s long-term growth or development interruption.

Given the complexity of economic system evolution, economic resilience still needs a unitary definition. Martin & Sunley (2015) proposed four widely recognized economic resilience dimensions, vulnerability, resistance, robustness, and recoverability [30]. From this, economic resilience refers to the ability of an economic system to withstand or recover from shocks to its previous development path, as well as to undergo adaptive changes in economic structures and institutional arrangements to transition to a new sustainable development way.

The present study summarizes the above content, as shown in Table 1:

**Table 1:** Comparison of two types of concept cognition of economic resilience.

Cognition perspective	Origin	Focus	Feature	Definition
Equilibrium-based perspective	Engineering resilience and ecological resilience	One ideal development state or multiple equilibrium states	Resistance and recoverability	The ability of an economic system to maintain normal function or gradually recover to the original development path when facing shocks.
Evolutionary perspective	Evolutionary resilience	No steady state, dynamic changes	Resistance, recoverability, and evolutionary	The ability of an economic system to withstand or recover from shocks to its previous development path, as well as to undergo adaptive changes in economic structures and institutional arrangements to transition to a new sustainable development way.

### Economic resilience evaluation

Previous studies employ descriptive and explanatory case studies, econometric models, indicator statistics, and other research methods to quantify economic resilience, and single-factor measurement and comprehensive indicator methods are the two most commonly used methods. The present study provides a review of the resilience surrogates method and index system method. These two methods represent the single-factor measurement method and the comprehensive indicator method, respectively.

#### Resilience surrogates method

Some scholars point out that economic resilience is the ability of economic entities to maintain structure and function during recessions and restore to pre-crisis levels. Therefore, capturing the resistance and recoverability of economic entities to shocks can measure economic resilience. The resilience surrogates method is constructed based on this principle. The resilience surrogates method reflects the strength of economic resilience by calculating the change degree of a specific factor before and after the shocks. The 2008 Financial Crisis is referred to as the crisis [19, 30], and the economic system’s cyclical rise and fall is referred to as the slow-moving disturbance [31, 32]. Calculation formulas are given as Eqs (1)–(3):

$${\left({\Delta \text{E}}_{\text{r}}^{\text{t}+\text{k}}\right)}^{\text{expected}}=\underset{\text{i}}{\sum }{\text{E}}_{\text{ir}}^{\text{t}}(1+{\text{g}}_{\text{N}}^{\text{t}+\text{k}})$$ (1)

$$\text{Resis}=\left[\Delta E_{r}^{\text{Contraction}}-{\left(\Delta E_{r}^{\text{Contraction}}\right)}^{\text{expected}}\right]/|{\left(\Delta E_{r}^{\text{Contraction}}\right)}^{\text{expected}}$$ (2)

$$\text{Recover}=\left[\Delta E_{r}^{\text{Recovery}}-{\left(\Delta E_{r}^{\text{Recovery}}\right)}^{\text{expected}}\right]/|{\left(\Delta E_{r}^{\text{Recovery}}\right)}^{\text{expected}}$$ (3)

In Eqs (1)–(3), ${\left(\Delta E_{r}^{t+k}\right)}^{expected}$ represents the expected growth of the surrogate variable during the k period, Resis represents the resistance dimension of economic resilience, and Recover represents the recoverability dimension of economic resilience. The movement between the actual and expected values of the surrogate variable expresses values of two dimensions. Generally, the resilience surrogates method mainly measures economic resilience’s resistance and recoverability dimensions.

Relevant research includes Brakman et al. (2015) measured the European countries’ economic resilience over the 2008 financial crisis by calculating the movement of GDP growth rate [19]. Bergeijk et al. (2017) chose foreign trade as a surrogate indicator to measure global countries’ economic resilience over the 2008 finance crisis [33]. Du et al. (2019) used GDP as a surrogate variable to measure the economic resilience of the Pearl River Delta [34]. Martin & Gardiner (2019) used the GDP growth rate to measure the resilience of cities facing economic shocks [35]. Feng et al. (2022) measured the economic resilience of urban agglomerations in China based on the movement of GDP growth rate [21].

#### Index system method

The index system method evaluates economic resilience through various indicators from multiple dimensions, which considers marginal contributions of more factors compared with the resilience surrogates method. This method is usually used in conjunction with indicator weighting and multi-attribute decision-making methods. In terms of dimension setting, there is no unified standard. Some articles divided dimensions based on the conceptual framework of economic resilience, such as resistance, recoverability, adaption, and creation. Some articles divided dimensions based on economic system structure, such as finance, enterprise, market, and governance.

Relevant research includes Wang et al. (2023) constructed an index system of economic resilience from the perspectives of economic performance, public health, epidemic management, and population information and applied it to the evaluation of 286 cities in China [36]. Jiang et al. (2022) established an index system for economic resilience based on five aspects: consumption, investment, import and export, government expenditure, and employment [37]. Huang et al. (2023) constructed an index system to evaluate urban economic resilience in three dimensions: resistance and recovery capacity, adaptation and adjustment capacity, and innovation and transformation capacity [16]. Ma & Huang (2023) evaluated the development level of economic resilience in three dimensions: resistance, recovery, and evolution [38].

The present study concludes the main evaluation methods of economic resilience, as shown in Table 2:

**Table 2:** Comparison of two types of evaluation methods of economic resilience.

Method	Category	Number of indicators	Advantage	Disadvantage	Relevant research	Indicator or dimension in common use
Resilience surrogates	Single-factor measurement method	One indicator	Easy to operate and compare horizontally	Focus on only one factor. The impact must reach a certain strength. Only measuring the dimensions of resistance and recoverability in economic resilience.	Brakman et al. (2015). Bergeijk et al. (2017). Du et al. (2019). Martin & Gardiner (2019). Feng et al. (2022)	Employment rate, unemployment rate, GDP, foreign trade.
Index system	Comprehensive indicator method	Multiple dimensions and indicators	Consider marginal contributions of more factors	Lack of unified standards for indicator selection and dimension setting. Causality confusion of indicators. The hierarchical structure of the index system is not clear.	Wang et al. (2023). Jiang et al. (2022). Huang et al. (2023). Ma & Huang (2023)	Resistance, recoverability, adaption, evolution, creation.

### Contribution of the present study

**Fig 1:**
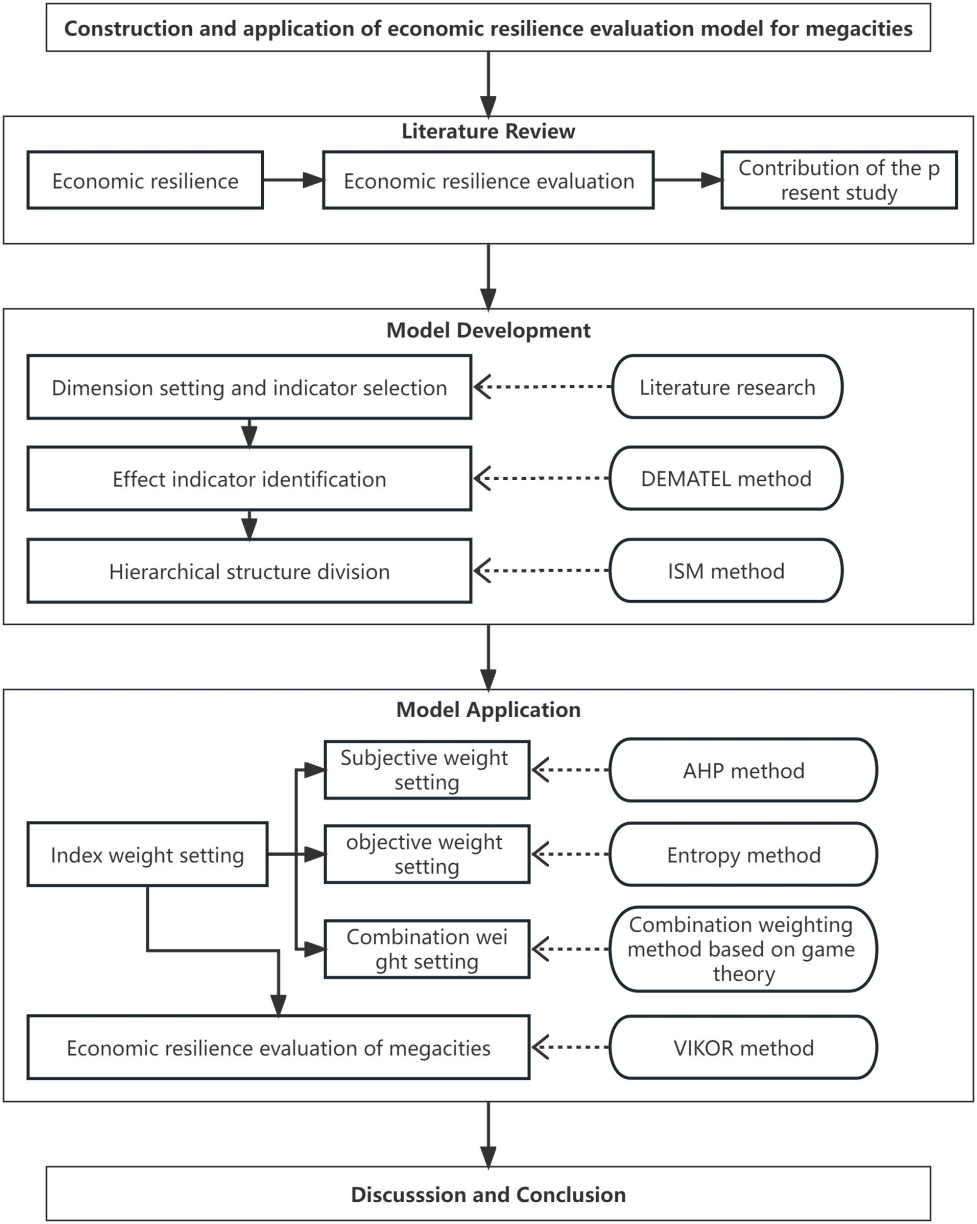
The workflow diagram of the study steps.

Previous studies have laid a preliminary foundation for the present study. The contribution of the present study mainly focuses on two aspects of research scale and methodology. In terms of research scale, previous studies explored the economic resilience of the EU scale, national scale, urban scale, rural scale, etc. Chinese scholars focus more on typical urban agglomerations, including the Yangtze River Economic Belt, Pearl River Delta urban agglomerations, Beijing-Tianjin-Hebei urban agglomerations, and the Guangdong-Hong Kong-Macao Greater Bay Area. The present study further expands the research scope of economic resilience from regional to urban areas, focusing on the economic resilience issues of megacities. In terms of methodology, the resilience surrogates method is convenient implementation and easy comparison, but it is challenging to consider the comprehensive impact of multiple factors. In addition, the shocks must reach a certain degree, leading to a significant change in the surrogate variables that can be used to measure economic resilience. The index system method considers the marginal contributions of more factors and evaluates economic resilience levels from multiple dimensions. However, there still lacks unified standards for the indicator selection and dimension setting, the causality of indicators is easily confused, and the hierarchical structure of the index system is also unclear. Since the index system method can compensate for the shortcomings of the resilience surrogates method to a certain extent. The second contribution of the present study is improving the drawbacks of the index system method and constructing a more accurate and effective evaluation model of the economic resilience of megacities. Moreover, the present study takes China’s megacities as a case study to apply the model and verify its effectiveness and reliability. The workflow diagram of the study steps is shown in Fig 1:

## Section 3: Evaluation model development of economic resilience

As an inherent attribute of the economic system, economic resilience is comprehensively affected by various factors and changes dynamically in the adaptive cycle [39]. The concept of economic resilience based on evolution theory has been more widely recognized, emphasizing the economic system’s adaptive and innovative capabilities to break through path dependence and transform it into a more efficient growth model. In order to consider more factors’ influence, the present study constructs an evaluation model of megacities based on the index system method. Before that, the present study uses comprehensive methods to overcome shortcomings of the index system method, including setting dimensions and indicators logically, identifying and excluding effect indicators, and dividing indicators’ hierarchy.

### Dimensions and indicators setting

The present study sets the evaluation dimensions and indicators based on acceptability, applicability, measurement, and availability principles.

Firstly, the present study determines the evaluation dimensions of economic resilience. The literature review shows that the division of evaluation dimensions varies from scholar to scholar. Some scholars set evaluation dimensions based on the subsystems’ functions, such as the financial and innovation subsystems; others set evaluation dimensions based on the characteristics of economic resilience, such as resistance, recoverability, and transformation. The present study learns from the previous research results to determine the evaluation dimensions of economic resilience. Specifically, the present study mainly refers to three classic theoretical research results of economic resilience: (i) Martin & Sunley (2010) borrowed the adaptive cycle model of resilience and divided the development cycle of the economic system into four stages: reorganization, development, conservation, and release. Economic resilience undergoes dynamic changes in four nested sub-cycles [39]; (ii) Davies (2011) built the conceptual model of economic resilience and set three dimensions: resistance, recoverability, and evolution, which respectively represent the ability of the economic system to resist external disturbances, recover to normal functions and create new development paths [40]; Martin & Sunley (2015) summarized four crucial dimensions of economic resilience: Vulnerability, resistance, robustness, and recoverability. These dimensions represent the capacity of the economic system to withstand or recover from shocks and, if necessary, undergo adaptive changes to transit to a new sustainable path [41]. With this in mind, the present study set three evaluation dimensions: Resistance, recoverability, and adaptability.

**Fig 2:**
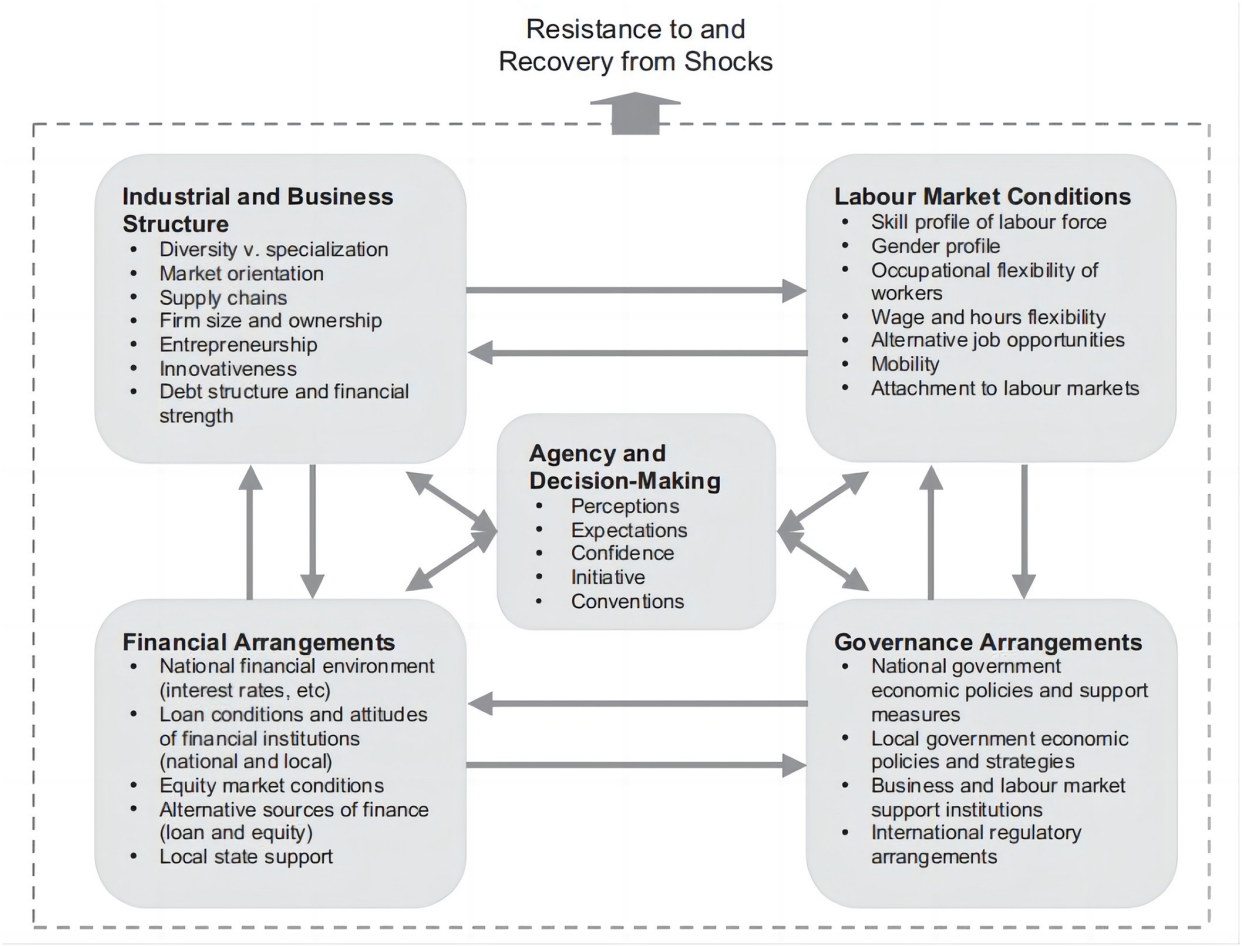
Determinants and framework of economic resilience [41].

Then, the present study sets evaluation indicators based on the literature review. (i) In theoretical research, the present study borrows the determinants and framework of economic resilience (Fig 2) developed by Martin & Sunley (2015). The framework has four aspects: Industrial and business structure, financial arrangements, governance arrangements, labour market conditions, and agency and decision-making. Each aspect contains several determinants of economic resilience. For example, the industrial and business structure aspect contains diversity v. specialization, market orientation, supply chains, etc. This framework is the primary basis of the indicator setting of the present study. (ii) In empirical research, the resilience surrogates method typically uses GDP, employment, unemployment, and foreign trade to measure economic resilience. In other words, these indicators are performance indicators of economic resilience, and the present study adopts them as evaluation indicators. The present study also learns from two famous relevant index systems. One is the Baseline Resilience Indicators for Communities (BRIC) index system proposed by Professor Cutter in 2010, and the other is the Resilience Capacity Index (RCI) index system submitted by the University at Buffalo. Since its implementation, the academic community and international organizations have widely recognized the BRIC index system. Because all indicators have been screened through factor analysis, and indicators’ data are from the public reports of the government or research institutions. BRIC index systems use indicators’ relative values for evaluation, which is suitable for horizontal comparison of multiple regions. The BRIC index systems regard economic resilience as one of the main dimensions and select four indicators to evaluate it: employment, the value of the property, wealth generation, and municipal finance/revenues. The RCI index system was developed to solve the problems of rapid population growth and systematic risk crises in metropolises, which is closely related to the research theme of this study. The RCI index system chooses four evaluation indicators for economic resilience: income equality, industrial diversification, purchasing power, and business environment.

In addition, more and more scholars use econometric models to identify influencing factors of economic resilience. The present study summarizes high-frequency and significant variables in literature, including Industrial diversification, innovation, openness, fiscal expenditure, human capital, financial development, informatization, and economic agglomeration.

Based on the discussion above, the present study develops the initial index system of economic resilience from three dimensions of resistance, recoverability, and adaptability, as shown in Table 3:

**Table 3:** Initial index system of economic resilience.

Dimension	Second-level indicator
Resistance	Economic foundation P1
	Employment P2
	Economic openness P3
	Social security P4
	Fixed assets investment P5
	Industrial scale P6
Recoverability	Economic agglomeration P7
	Self-financing P8
	Market potential P9
	Financial development P10
	Industry diversity P11
	Informationalization P12
Adaptability	R&D investment P13
	Innovation output P14
	Entrepreneurial activity P15
	Human capital quality P16
	Industrial structure advancement P17
	New economic sectors development P18

These indicators are divided into two categories according to the data resource: Statistical and computational indicators. The statistical indicators obtain data directly from the yearbook or official documents published by the national or local governments, and the computational indicators receive data through equation calculation.

### Effect indicator identification

Index system method used to evaluate economic resilience are widely questioned due to the unclear causality of indicators. Specifically, the index system may include cause and effect indicators at the same time. The effect indicator is the outcome of economic resilience improvement, which should be excluded from the index system. Therefore, the present study employs the DEMATEL method to identify and exclude effect indicators from the initial index system.

The DEMATEL method uses the matrix tool to calculate the influence degree between indicators to quantify the interrelationship among the evaluation indicators [42, 43]. Influence degree is used to calculate the centrality of each indicator, which can distinguish the effect indicators. The application process of the DEMATEL method is as follows:

Firstly, the authors invite several experts in the field to judge the impact relationship between the 18 secondary indicators in the initial index system of economic resilience. The experts use the five-level scale to quantify the indicators’ influence: Extremely strong (4), strong (3), moderate (2), weak (1), and no impact (0). Every two indicators need to be compared twice. For example, indicator P_i_ should be compared twice with indicator P_j_. One is the direct impact of P_i_ on P_j_, and the other is the direct impact of P_j_ on P_i_. Then, the direct relation matrix is obtained based on expert scoring, as expressed by Eq (4):

$$\text{B}=\left[\begin{array}{ccc} \begin{array}{c} 0\\ {\text{b}}_{21} \end{array} & \begin{array}{c} {\text{b}}_{12}\\ 0 \end{array} & \begin{array}{cc} \begin{array}{c} \cdots \\ \cdots \end{array} & \begin{array}{c} {\text{b}}_{1\text{j}}\\ {\text{b}}_{21} \end{array} \end{array}\\ \vdots & \ddots & \vdots \\ {\text{b}}_{\text{i}1} & {\text{b}}_{\text{i}2} & \begin{array}{cc} \cdots & 0 \end{array} \end{array}\right]$$ (4)

Then, the total relation matrix can be obtained, as expressed by Eq (5):

$$T=C+C^{2}+\cdots +C^{n}=C\frac{I-C^{n-1}}{I-C}$$ (5)

In Eq (5), I represent the identity matrix, and 0 ≤ c_ij_ ≤ 1. When n → ∞, C^n-1^ → 0. Therefore, Eq (5) is converted to Eq (6):

$$T=C{\left(I-C\right)}^{-1}$$ (6)

Then, the present study calculated each indicator’s influence and affected degree, which is represented by R and C, respectively, as shown in Eqs (7) and (8):

$$R=\overset{n}{\underset{j=1}{\sum }}t_{ij},\;i=1,2,\cdots ,n$$ (7)

$$C=\overset{n}{\underset{j=1}{\sum }}t_{ij},\;j=1,2,\cdots ,n$$ (8)

Then, the present study calculates each indicator’s centrality and cause degree, which M and N represent, respectively, as shown in Eqs (9) and (10):

M=R+C (9)

N=R−C (10)

If N > 0, the corresponding indicator is the cause indicator. Otherwise, this indicator is the effect indicator.

The cause-effect analysis of indicators is shown in Table 4:

**Table 4:** Cause-effect analysis of indicators.

Indicator	R	C	M	N	Type
Economic foundation P1	4.3005	4.2860	8.5865	0.0145	Cause
Employment P2	3.4181	3.3516	6.7697	0.0665	Cause
Economic openness P3	3.5968	3.5903	7.1871	0.0065	Cause
Social security P4	2.9683	2.7542	5.7225	0.2141	Cause
Fixed assets investment P5	2.7869	2.6852	5.4721	0.1017	Cause
Industrial scale P6	3.4091	3.3559	6.7650	0.0533	Cause
Economic agglomeration P7	3.2664	3.1280	6.3944	0.1383	Cause
Self-financing P8	3.5399	3.5092	7.0491	0.0307	Cause
**Market potential P9**	**2.7344**	**3.1518**	**5.8862**	**-0.4174**	**Effect**
Financial development P10	3.2806	3.2623	6.5429	0.0183	Cause
Industry diversity P11	3.4938	3.4825	6.9763	0.0113	Cause
Informationalization P12	3.2068	3.1809	6.3877	0.0259	Cause
R&D investment P13	3.7207	3.6000	7.3207	0.1207	Cause
Innovation output P14	3.5034	3.4635	6.9669	0.0399	Cause
Entrepreneurial activity P15	3.2275	3.2147	6.4421	0.0128	Cause
Human capital quality P16	3.5147	3.4669	6.9816	0.0477	Cause
**Industrial structure advancement P17**	**3.4506**	**3.4806**	**6.9312**	**-0.0300**	**Effect**
**New economic sectors development P18**	**2.8784**	**3.3331**	**6.2115**	**-0.4547**	**Effect**

The initial index system contains three effect indicators: Market potential P9, industrial structure advancement P17, and new economic sectors development P18. The primary reasons are as follows: when the megacity has strong economic resilience, residents are confident in the government and policies, and they tend to reduce savings and increase consumption, which enhances the market potential. Besides, high-level economic resilience promotes the megacity to optimize the economic structure and seek new economic growth paths. During this process, the city gradually formed an advanced industrial structure and new economic sectors. In summary, market potential, industrial structure advancement, and new economic sectors development result in economic resilience improvement, not its causes.

Therefore, the present study establishes the secondary index system, as shown in Table 5:

**Table 5:** Secondary index system of economic resilience.

Dimension	Second-level indicator
Resistance	Economic foundation S1
	Employment S2
	Economic openness S3
	Social security S4
	Fixed assets investment S5
	Industrial scale S6
Recoverability	Economic agglomeration S7
	Self-financing S8
	Financial development S9
	Industry diversity S10
	Informationalization S11
Adaptability	R&D investment S12
	Innovation output S13
	Entrepreneurial activity S14
	Human capital quality S15

### Hierarchical structure division

The secondary index system has three dimensions and 15 second-level indicators, and three effect indicators have been removed. However, second-level indicators have not been divided into levels. More empirical evidence should be provided to classify second-level indicators and sort them into the corresponding dimensions.

The present study uses the ISM method to divide the hierarchical structure of second-level indicators. Professor Warfield first proposed the ISM method, which was used to conduct a hierarchical analysis of the socioeconomic system [44]. ISM method decomposes a complex system into several subsystems and derives their connection structure based on subjective judgment, practical experience, and matrix tools. Then, the complex system is transformed into a clear and multi-level hierarchical structure. Similar to the DEMATEL method, the ISM method also considers the interrelationship between indicators and divides the hierarchical structure by constructing and solving the reachability matrix to explain the order, direction, and complex relationship of different indicators in the system. The process of the ISM method is as follows:

Firstly, the present study establishes the adjacency matrix, as shown in Eq (11):

$$\bar{\text{H}}\in \left\{\begin{array}{ll} 1, & h_{ij}\geq \lambda \\ 0, & h_{ij}<\lambda \end{array}\right.$$ (11)

Then, the reachability matrix is obtained as shown in Eq (12):

$$\text{M}={\left(\bar{\text{H}}\right)}^{\text{k}}={\left(\bar{\text{H}}\right)}^{\text{k}-1}\neq {\left(\bar{\text{H}}\right)}^{\text{k}-2}\neq \cdots \neq \bar{\text{H}},\;\;\text{k}\leq \text{n}-1$$ (12)

Then, the hierarchical structure is obtained based on the reachability matrix, which is expressed by Eq (13):

$$\text{C}\left(S_{i}\right)=\left\{{\text{P}}_{\text{i}}|R\left({\text{S}}_{\text{i}}\right)\cap A\left({\text{S}}_{\text{i}}\right)=R\left({\text{S}}_{\text{i}}\right),\;\;\text{i}=1,2,\cdots ,\;\;\text{n}\right\}$$ (13)

R(S_i_) represents a reachable set, A(S_i_) represents an antecedent set, and C(S_i_) represents a common set. The result is shown in Table 6:

**Table 6:** Reachable set, antecedent set, and a common set.

No.	Reachable set	Antecedent set	Common set
1	1	1, 2, 3, 5, 6, 7, 9, 10, 11, 12, 13, 14, 15	1
2	1, 2, 8	2	2
3	1, 3	3, 7, 9, 11, 13	3
4	4	4	4
5	1, 5	5	5
6	1, 6	6	6
7	1, 3, 7, 10	7	7
8	8	2, 8, 11	8
9	1, 3, 9	9	9
10	1, 10	7, 10, 11	10
11	1, 3, 8, 10, 11, 12, 13, 14, 15	11	11
12	1, 12	11, 12, 14, 15	12
13	1, 13	11, 13	13
14	1, 3, 12, 14	11, 14	14
15	1, 12, 15	11, 15	15

The hierarchical structure of 15 second-level indicators is shown in Table 7:

**Table 7:** The hierarchical structure of second-level indicators.

Level	Indicator
L1	S1, S4, S8
L2	S2, S3, S5, S6, S10, S12, S13
L3	S7, S9, S14, S15
L4	S11

Table 7 shows that 15 second-level indicators are divided into four layers. There are three first-level indicators, and the L4 level only has one. Therefore, the present study mergers the L3 and L4 level, and the hierarchical structure are as follows:

The L1 level represents the resistance dimension of economic resilience and contains three second-level indicators: Economic level S1, social security level S4, and self-financing level S8.

The L2 level represents the recoverability dimension of economic resilience. It contains seven second-level indicators: Employment rate S2, economic openness degree S3, fixed assets investment S5, industrial scale S6, industry diversity degree S10, R&D investment S12, and innovation output S13.

The L3 and L4 levels merge into one layer, representing the adaptability dimension. This layer contains five second-level indicators: Economic agglomeration level S7, financial development level S9, informationalization level S11, entrepreneurial activity S14, and human capital quality S15.

The present study renumbers 15 second-level indicators according to their hierarchical structure and establishes the final evaluation index system, as shown in Table 8:

**Table 8:** Final evaluation index system of economic resilience.

Dimension	Second-level indicator
Resistance	Economic foundation Q1
	Employment Q2
	Economic openness Q3
Recoverability	Social security Q4
	Fixed assets investment Q5
	Industrial scale Q6
	Economic agglomeration Q7
	Self-financing Q8
	Financial development Q9
	Industry diversity Q10
Adaptability	Informationalization Q11
	R&D investment Q12
	Innovation output Q13
	Entrepreneurial activity Q14
	Human capital quality Q15

## Section 4: Model application

The present study takes Chinese megacities as a case study to validate the model’s effectiveness. The data comes from megacities’ *City Statistical Yearbook* and *Statistics Communique on National Economy and Social Development*. In addition, some data need to be taken from the *China Industrial Enterprises Database*, *China City Statistical Yearbook*, and *City Construction Statistical Yearbook*.

### Overview of Chinese megacities

Megacity refers to a city with more than 10 million permanent residents who have lived in the urban area for more than half a year. According to China’s seventh national population census, there are seven megacities currently according to the Seventh Population Census of China, including Beijing, Tianjin, Shanghai, Guangzhou, Shenzhen, Chongqing, and Chengdu. The present study uses these seven megacities to apply the evaluation model of economic resilience mentioned above. Since the Seventh Population Census in China was conducted from November to December 2020, the present study focuses on the research period of 2011–2020. Detail is shown in Table 9:

**Table 9:** Existing megacities in China (Data from China’s seventh population census).

City	Urban permanent population (10000 people)	City level	Region
Beijing	1775	Capital, municipality	North China
Tianjin	1093	Municipality	North China
Shanghai	1987	Municipality	East China
Guangzhou	1488	Provincial capital	South China
Shenzhen	1744	Special economic zone	South China
Chongqing	1634	Municipality	Southwest China
Chengdu	1334	Provincial capital	Southwest China

**Fig 3:**
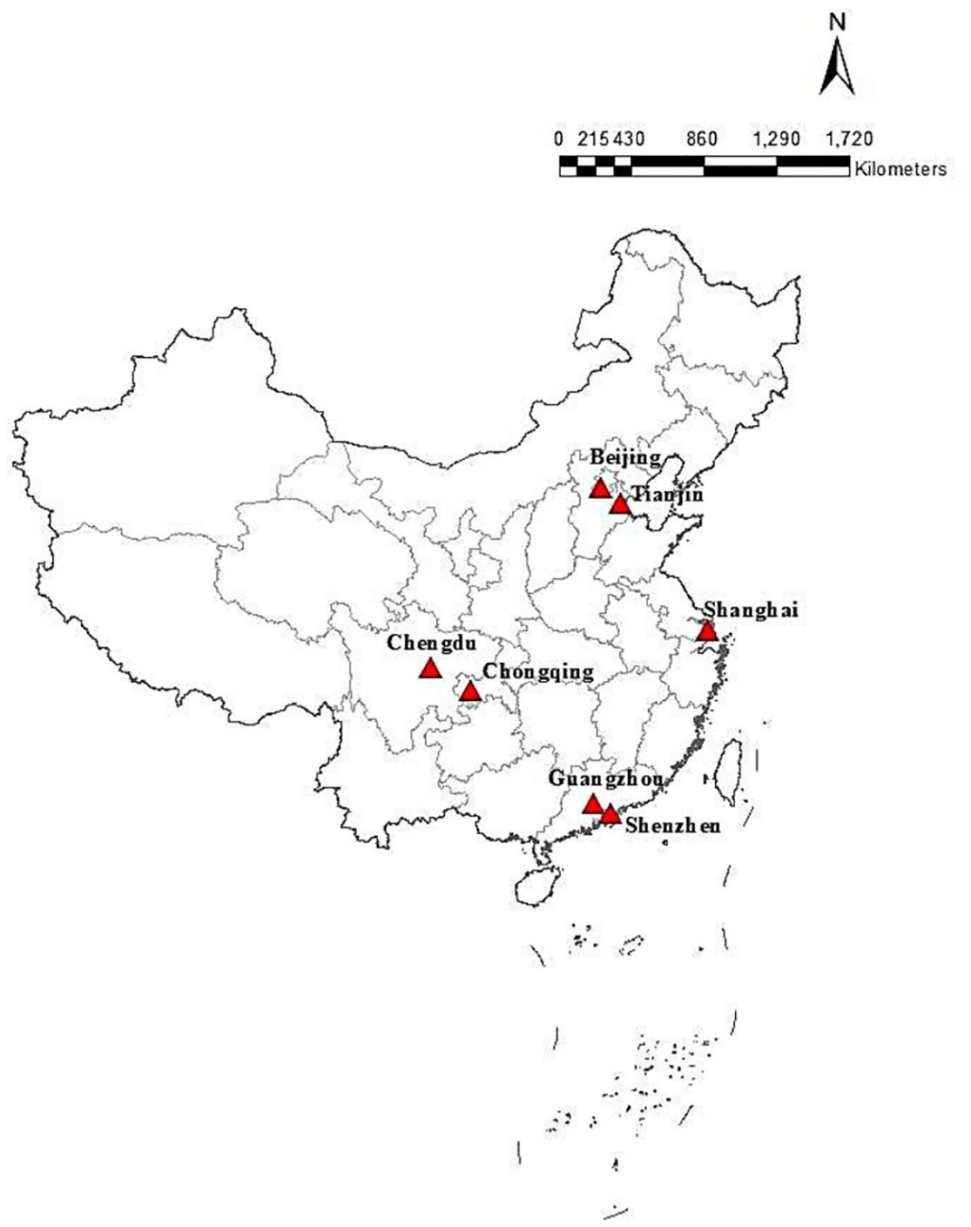
Geographical location distribution of megacities in China.

The location of the mentioned megacities is shown in Fig 3:

As the highest-ranked and most populous city in the urban system, megacities are the economic, political, technological innovation, and cultural centers of the region and even the entire country. Among them, Beijing, Shanghai, Guangzhou, and Shenzhen are global cities that consistently influence on world economic affairs. Megacities are of great significance for the development of China’s national economy.

Firstly, from the perspective of economic scale, the total GDP of megacities accounts for an absolute proportion of the national GDP (about 18%). The details are shown in Table 10.

**Table 10:** Total GDP and proportion of mega cities in 2011–2020 (Unit: Trillion).

	2011		2012		2013		2014		2015	
	GDP	Proportion	GDP	Proportion	GDP	Proportion	GDP	Proportion	GDP	Proportion
Beijing	1.72	3.56%	1.90	3.54%	2.11	3.59%	2.29	3.55%	2.48	3.62%
Tianjin	0.81	1.68%	0.90	1.67%	0.99	1.68%	1.06	1.64%	1.09	1.59%
Shanghai	2.00	4.14%	2.13	3.96%	2.32	3.94%	2.53	3.93%	2.69	3.92%
Guangzhou	1.22	2.52%	1.32	2.46%	1.51	2.57%	1.61	2.50%	1.73	2.52%
Shenzhen	1.19	2.46%	1.35	2.51%	1.52	2.58%	1.68	2.61%	1.84	2.68%
Chongqing	1.02	2.11%	1.16	2.16%	1.30	2.21%	1.46	2.27%	1.60	2.33%
Chengdu	0.73	1.51%	0.86	1.60%	0.95	1.62%	1.04	1.61%	1.07	1.56%
**Total**	**8.69**	**17.98%**	**9.62**	**17.90%**	**10.7**	**18.19%**	**11.67**	**18.11%**	**12.5**	**18.23%**
	2016		2017		2018		2019		2020	
	GDP	Proportion	GDP	Proportion	GDP	Proportion	GDP	Proportion	GDP	Proportion
Beijing	2.70	3.64%	2.99	3.60%	3.31	3.62%	3.54	3.60%	3.59	3.56%
Tianjin	1.15	1.55%	1.25	1.50%	1.34	1.46%	1.41	1.43%	1.40	1.39%
Shanghai	2.99	4.03%	3.29	3.96%	3.60	3.93%	3.80	3.86%	3.90	3.87%
Guangzhou	1.86	2.50%	1.99	2.39%	2.10	2.29%	2.36	2.40%	2.51	2.49%
Shenzhen	2.07	2.79%	2.33	2.80%	2.53	2.76%	2.69	2.73%	2.78	2.76%
Chongqing	1.80	2.42%	2.01	2.42%	2.16	2.36%	2.36	2.40%	2.50	2.48%
Chengdu	1.19	1.60%	1.39	1.67%	1.57	1.72%	1.70	1.73%	1.78	1.76%
**Total**	**13.76**	**18.53%**	**15.25**	**18.35%**	**16.61**	**18.15%**	**17.86**	**18.15%**	**18.46**	**18.30%**

**Fig 4:**
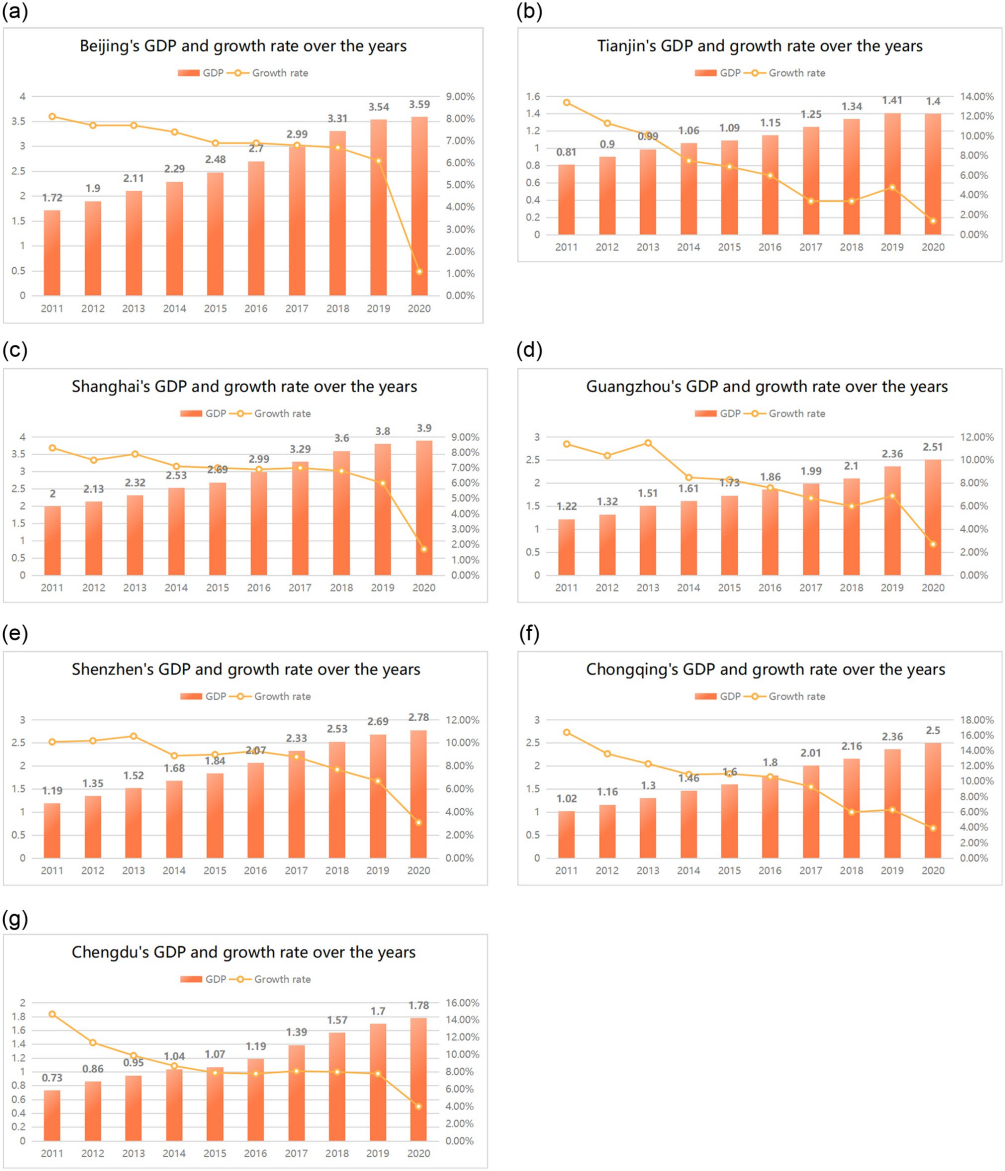
(a). Beijing’s GDP and growth rate. (b). Tianjin’s GDP and growth rate. (c). Shanghai’s GDP and growth rate. (d). Guangzhou’s GDP and growth rate. (e). Shenzhen’s GDP and growth rate. (f). Chongqing’s GDP and growth rate. (g). Chengdu’s GDP and growth rate.

Secondly, from the perspective of development trends, each megacity’s GDP has been increasing year by year, as shown in Fig 4(a)–4(g):

Finally, from the perspective of economic status, megacities are the main driving force for the economic development of respective regions.

Beijing and Tianjin are the central cities of the Beijing-Tianjin-Hebei Urban Agglomeration. *The Outline of the Beijing-Tianjin-Hebei Collaborative Development Plan* proposes the formation of an urban agglomeration distribution structure of “one core, two cities, three axes, four districts, and multiple nodes.” The “one core” refers to prioritizing the enhancement of Beijing’s core functions, and the “two cities” refers to leveraging the leading engine role of Beijing and Tianjin, promoting the coordinated development of the entire urban agglomeration through high-end leadership and radiation.

Shanghai is the central city of the Yangtze River Delta urban agglomeration. The Yangtze River Delta Urban Agglomeration is currently the most significant urban agglomeration in China’s economy and cities, with a GDP exceeding one trillion yuan, accounting for over 30% of the national total. The internal hierarchical structure of this urban agglomeration is the most reasonable. With its high-end manufacturing industry, modern service industry, and new economy sectors, Shanghai has played a decisive role in resource aggregation and radiation leadership for the region.

Guangzhou and Shenzhen are the central cities of the Pearl River Delta Urban Agglomeration. The private economy in this urban agglomeration is the most active, especially in the trade and labor-intensive industries, which have attracted a large number of foreign populations. Guangzhou and Shenzhen have advantageous sectors, such as new energy vehicles, smart home appliances, and consumer electronics, with great potential for economic structural transformation and upgrading. Compared to Beijing and Shanghai, the threshold for settling down is relatively low, and there has been a phenomenon of “population inversion.”

Chongqing and Chengdu are the central cities of the Chengdu Chongqing Economic Circle, and both were selected as national central cities in 2016. Chongqing mainly plays the financial center role, with highly developed modern service industries such as finance, commerce, logistics, and service outsourcing. Chengdu is a technology innovation center with multiple high-tech entrepreneurship bases. Chongqing and Chengdu have led the economic development of the economic circle and the entire Southwest region.

### Index weight setting

Standard indicator weighting methods are mainly divided into three categories: subjective, objective, and combination weighting methods. Subjective weighting methods include AHP, Delphi, Analytic Network Process (ANP), etc. Objective weighting methods include Principal component analysis (PCA), Entropy, Multi-objective programming (MOP), etc. The combination weighting methods combine the subjective and objective weighting methods, which overcome shortcomings arising from the independent use of the subjective or objective weighting method.

Therefore, the present study uses the combination weighting method based on game theory to calculate the indicators’ weights. This method seeks a balanced solution between subjective and objective weight. The former is calculated by the AHP method, and the latter is calculated by the Entropy method.

#### Subjective weight setting

The present study uses the AHP method to calculate the indicators’ subjective weights. The AHP method was first proposed by Professor Saaty of the University of Pittsburgh in 1970. Its principle is to decompose a systematic decision-making problem into several levels of sub-objectives, sub-criteria, or sub-constraints and use the fuzzy quantitative method to solve the single ranking of each level and the total ranking. Specifically, the AHP method is to decompose a multi-objective decision-making problem into target level, criterion level, and scheme level and use an expert scoring method to determine the importance order of the indicators at the scheme level to obtain the judgment matrix and solve the subjective weight of all indicators at the scheme level by calculating the matrix eigenvalue and eigenvector, and then use the weighted sum method of hierarchical merging to obtain the indicator weights at the criterion level and the target level in turn. The specific process is as follows:

Firstly, the present study establishes the judgment matrix that is expressed by Eq (14):

$$C_{u}={\left(c_{ij}\right)}_{n\times n}=\left(\begin{array}{ccc} c_{11} & \cdots & c_{1n}\\ \vdots & \ddots & \vdots \\ c_{n1} & \cdots & c_{nn} \end{array}\right)$$ (14)

c_ij_ represents the importance comparison value between indicators i and j, and it is obtained by the 1–9 scale method, as shown in Table 11:

**Table 11:** The 1–9 scale method of the analytic hierarchy process.

Importance comparison	Value
Indicator i is as important as j	1
Indicator i is slightly more important than j	3
Indicator i is obviously more important than j	5
Indicator i is enormously more important than j	7
Indicator i is hugely more important than j	9
An intermediate value of two adjacent judgment results	2, 4, 6, 8

Then, the present study calculates the maximum eigenvalue of the judgment matrix and tests its consistency. Consistency test equations are shown in Eqs (15) and (16):

$$CI=\frac{\lambda_{max}-n}{n-1}$$ (15)

$$CR=\frac{CI}{RI}$$ (16)

If CR < 0.1, the maximum eigenvalue passes the consistency test, and the subjective weight is expressed by Eq (17):

$$\xi_{k}={\left(I_{1k},I_{2k},\cdots ,I_{nk}\right)}^{T}$$ (17)

Based on Eqs (11)–(13), the present study obtains all indicators’ subjective weight, as shown in Table 12:

**Table 12:** Indicators’ subjective weight based on the AHP method.

Dimension	Second-level indicator	Subjective weight
Resistance (0.491)	Economic foundation Q1	0.260
	Employment Q2	0.075
	Economic openness Q3	0.156
Recoverability (0.393)	Social security Q4	0.069
	Fixed assets investment Q5	0.079
	Industrial scale Q6	0.077
	Economic agglomeration Q7	0.071
	Self-financing Q8	0.028
	Financial development Q9	0.042
	Industry diversity Q10	0.027
Adaptability (0.114)	Informationalization Q11	0.020
	R&D investment Q12	0.038
	Innovation output Q13	0.023
	Entrepreneurial activity Q14	0.019
	Human capital quality Q15	0.014

#### Objective weight setting

The present study uses the entropy method to calculate the indicators’ objective weight. The essence of the entropy method is information entropy, which calculates the difference and dispersion degree of different evaluation indicators as their weights. The Entropy method can comprehensively reflect all the data information of the evaluation indicators, and it is scientific and reasonable when solving the objective weight.

The entropy method is commonly applied in handling time series data, and the present study adds time variables into the traditional entropy method, called the improved entropy method. Thus, the improved entropy method can be applied to handling panel data. The specific process is as follows:

Firstly, the present study normalizes the panel data and calculates the indicators’ information utility value, as shown in Eqs (18) and (19):

$$P_{\alpha ij}=\frac{{Q^{\prime }}_{\alpha ij}}{\sum_{\alpha =1}^{10}\sum_{i=1}^{7}{Q^{\prime }}_{\alpha ij}}$$ (18)

$$E_{j}=-k\overset{10}{\underset{\alpha =1}{\sum }}\overset{7}{\underset{i=1}{\sum }}P_{\alpha ij}ln(P_{\alpha ij})$$ (19)

Then, the present study calculates the redundancy and objective weight of each indicator, as shown in Eqs (20) and (21):

$$D_{j}=1-E_{j}$$ (20)

$$W_{j}=\frac{D_{j}}{\sum_{j=1}^{n}D_{j}}$$ (21)

Based on the process, the results are shown in Table 13:

**Table 13:** The indicators’ objective weight.

Dimension	Second-level indicator	Information entropy	Redundancy	Objective weight
Resistance (0.196)	Economic foundation Q1	20.597	-19.597	0.044
	Employment Q2	46.234	-45.234	0.101
	Economic openness Q3	23.948	-22.948	0.051
Recoverability (0.501)	Social security Q4	45.843	-44.843	0.101
	Fixed assets investment Q5	15.660	-14.660	0.033
	Industrial scale Q6	55.741	-54.741	0.123
	Economic agglomeration Q7	36.166	-35.166	0.079
	Self-financing Q8	34.149	-33.149	0.074
	Financial development Q9	19.280	-18.280	0.041
	Industry diversity Q10	23.461	-22.461	0.050
Adaptability (0.303)	Informationalization Q11	5.809	-4.809	0.011
	R&D investment Q12	26.813	-25.813	0.058
	Innovation output Q13	35.286	-34.286	0.077
	Entrepreneurial activity Q14	34.355	-33.355	0.075
	Human capital quality Q15	37.467	-36.467	0.082

#### Combination weight setting

The present study uses the combination weighting method based on game theory to calculate the total weight of each indicator. This method’s nature is seeking a compromise solution between the subject and object weight, as shown in Eq (22):

$$w_{j}=t_{1}\alpha_{j}+t_{2}\beta_{j}\;\left(j=1,2,\cdots ,n\right)$$ (22)

In Eq (22), w_j_ represents the total weight, α_j_ represents the subjective weight, and β_j_ represents the objective weight.

Based on the differential property of the matrix, the optimal first derivative condition of the above weight vector optimization model can be solved, which needs to meet the following:

$$\left(\begin{array}{cc} \alpha \alpha^{T} & \beta \alpha^{T}\\ \alpha \beta^{T} & \beta \beta^{T} \end{array}\right)\left(\begin{array}{c} t_{1}\\ t_{2} \end{array}\right)=\left(\begin{array}{c} \alpha \alpha^{T}\\ \beta \beta^{T} \end{array}\right)$$ (23)

Based on the Eqs (22) and (23), the indicators’ total weight is shown in Table 14:

**Table 14:** The indicators’ total weight.

Dimension	Second-level indicator	Total weight
Resistance (0.349)	Economic foundation Q1	0.156
	Employment Q2	0.088
	Economic openness Q3	0.105
Recoverability (0.445)	Social security Q4	0.084
	Fixed assets investment Q5	0.057
	Industrial scale Q6	0.099
	Economic agglomeration Q7	0.075
	Self-financing Q8	0.050
	Financial development Q9	0.042
	Industry diversity Q10	0.038
Adaptability (0.206)	Informationalization Q11	0.016
	R&D investment Q12	0.048
	Innovation output Q13	0.049
	Entrepreneurial activity Q14	0.046
	Human capital quality Q15	0.047

### Economic resilience evaluation

The present study uses seven Chinese megacities to show the model application. Because the evaluation of economic resilience is a relative comparison between megacities, similar to the multiple alternatives ranking, and the present study adopts the VIKOR method to deal with the final economic resilience evaluation index system in Table 8.

The VIKOR method was first proposed by Professor Opricovic in 1998 and is a multi-attribute decision-making method based on an ideal solution. The main principle of the VIKOR method is to find the positive ideal solution (the best performance based on the evaluation criteria) and the negative ideal solution (the worst performance based on the evaluation criteria) of all candidate solutions. It decides between the positive and the negative ideal solution. Then, the candidate schemes are ranked by comparing the difference between the evaluation value of each candidate scheme and the ideal solution. Therefore, the VIKOR method is an aggregation function developed on the Lp-metric aggregate function, as shown in Eq (24):

$$L_{pj}={\left[\sum_{i=1}^{n}{\left[W_{j}\left(f_{i}^{+}-f_{ij}\right)\left(f_{i}^{+}-f_{i}^{-}\right)\right]}^{p}\right]}^{1/p}$$ (24)

In Eq (24), L_pj_ represents the distance from each candidate solution to the ideal solution, f_ij_ represents the evaluation value of each indicator, and $f_{i}^{+}$ and $f_{i}^{-}$ represent the positive and negative ideal solution, respectively. p represents the distance parameter of the aggregation function, and W_j_ represents the evaluation indicator’s weight.

Firstly, the present study calculates indicators’ positive and negative ideal solutions, as shown in Eqs (25) and (26):

$$f_{j}^{*}=\left[\left(\left.\underset{i}{\text{max}}f_{ij}\right|j\in I_{1}\right),\;\left(\left.\underset{i}{\text{min}}f_{ij}\right|j\in I_{2}\right)\right]\forall j$$ (25)

$$f_{j}^{-}=\left[\left(\left.\underset{i}{\text{min}}f_{ij}\right|j\in I_{1}\right),\;\left(\left.\underset{i}{\text{max}}f_{ij}\right|j\in I_{2}\right)\right]\forall j$$ (26)

Then, the present study obtains the group utility valve and individual regret value, as shown in Eqs (27) and (28):

$$S_{i}=\overset{n}{\underset{j=1}{\sum }}w_{j}\frac{\left(f_{j}^{*}-f_{ij}\right)}{\left(f_{j}^{*}-f_{j}^{-}\right)}$$ (27)

$$R_{i}=\underset{j}{\text{max}}w_{j}\frac{\left(f_{j}^{*}-f_{ij}\right)}{\left(f_{j}^{*}-f_{j}^{-}\right)}$$ (28)

Based on Eqs (27) and (28), the compromise value is expressed by Eq (29):

$$V_{i}=\nu \frac{S_{i}-{\text{S}}^{*}}{{\text{S}}^{*}-{\text{S}}^{-}}+\left(1-\nu \right)\frac{R_{i}-{\text{R}}^{*}}{{\text{R}}^{*}-{\text{R}}^{-}}$$ (29)

Because the smaller the compromise value is, the better the alternative is, and the economic resilience level is expressed by Eq (30):

$$U_{\text{ER}}=1-\text{V}$$ (30)

The present study chooses seven megacities in China as examples, and their economic resilience evaluation results are shown in Table 15:

**Table 15:** Evaluation results of megacities’ economic resilience in 2011–2020.

	**2011**	**2012**	**2013**	**2014**	**2015**
**Beijing**	1.0000	1.0000	1.0000	1.0000	1.0000
**Tianjin**	0.6421	0.5438	0.6049	0.6414	0.6510
**Shanghai**	0.8818	0.8231	0.7711	0.7965	0.8720
**Guangzhou**	0.6703	0.5986	0.6269	0.6021	0.6005
**Shenzhen**	0.5795	0.6186	0.7081	0.7762	0.7759
**Chongqing**	0.0000	0.0295	0.0587	0.0896	0.1350
**Chengdu**	0.2587	0.1570	0.1869	0.1749	0.1718
	**2016**	**2017**	**2018**	**2019**	**2020**
**Beijing**	0.9812	0.9698	0.8946	0.8210	0.8035
**Tianjin**	0.6225	0.5000	0.5536	0.5749	0.2980
**Shanghai**	0.8014	0.9170	0.9113	0.8512	0.8319
**Guangzhou**	0.5613	0.5139	0.5486	0.5000	0.5540
**Shenzhen**	0.6413	0.7593	0.8529	0.9174	0.9319
**Chongqing**	0.1491	0.1400	0.1224	0.1830	0.1413
**Chengdu**	0.1369	0.2014	0.2093	0.3265	0.2408

The megacities’ economic resilience are all in the range of 0 and 1 based on the VIKOR method. Therefore, the present study proposed the classification criteria, as shown in Table 16:

**Table 16:** Classification criteria of megacities’ economic resilience level.

Economic resilience level	Evaluation value
Highest	0.80 < U_ER_ ≤ 1.00
Higher	0.60 < U_ER_ ≤ 0.80
Middle	0.40 < U_ER_ ≤ 0.60
Lower	0.20 < U_ER_ ≤ 0.40
Lowest	0.00 < U_ER_ ≤ 0.20

Based on Table 16, the present study partitions seven megacities in China into different layers according to their economic resilience level, as shown in Table 17:

**Table 17:** Economic resilience level distribution of megacities in China during 2011–2020.

**Economic resilience level**	**2011**	**2012**	**2013**	**2014**	**2015**
**Highest**	2	2	1	1	2
**Higher**	2	1	4	4	3
**Middle**	1	2	0	0	0
**Lower**	1	0	0	0	0
**Lowest**	1	2	2	2	2
**Highest and a higher proportion**	57.14%	42.86%	71.43%	71.43%	71.43%
	**2016**	**2017**	**2018**	**2019**	**2020**
**Highest**	2	2	3	3	3
**Higher**	2	1	0	0	0
**Middle**	1	2	2	2	1
**Lower**	0	1	1	1	2
**Lowest**	2	1	1	1	1
**Highest and a higher proportion**	57.14%	42.86%	42.86%	42.86%	42.86%

**Fig 5:**
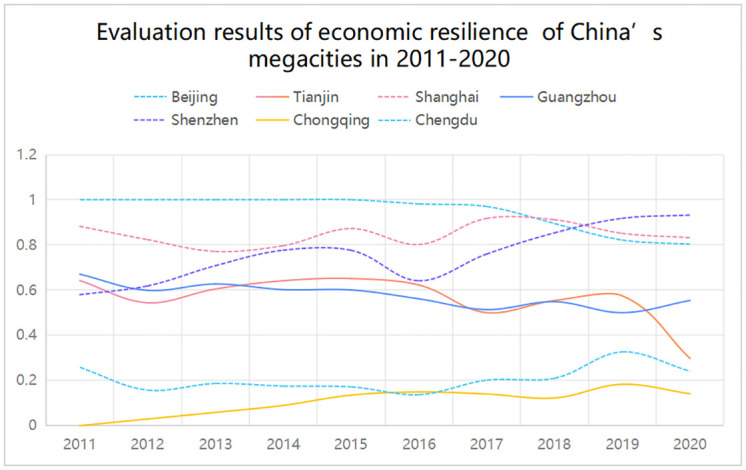
Evaluation results of economic resilience of China’s megacities in 2011–2020.

**Fig 6:**
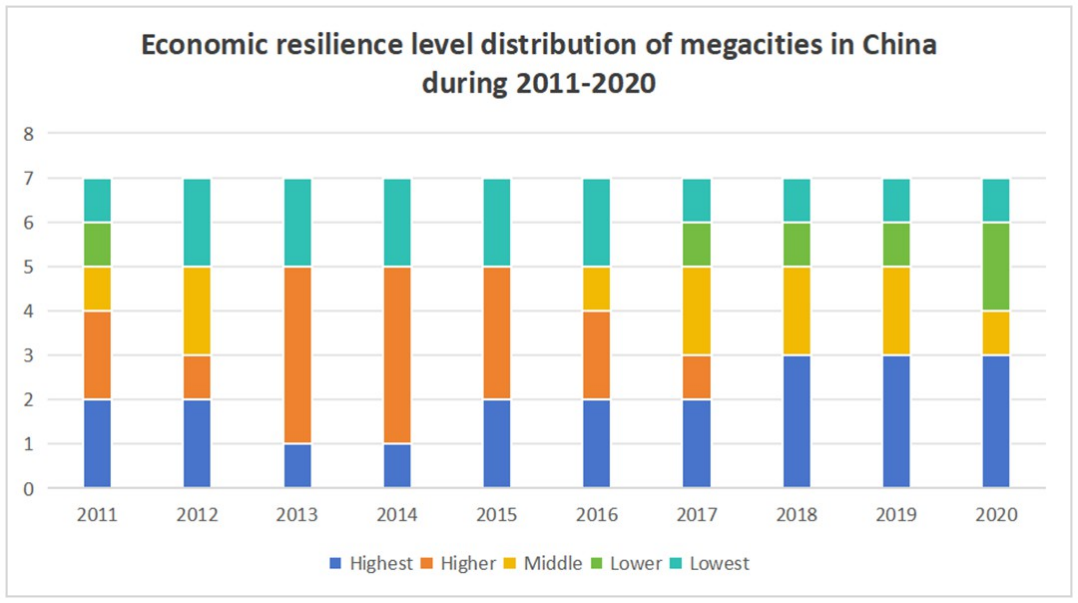
Economic resilience level distribution of megacities in China during 2011–2020.

The present study summarizes the content of Tables 15 and 17, as shown in Figs 5 and 6:

The main conclusions are as follows:

Firstly, after evaluating the economic resilience of seven megacities in China, it was found that from 2010 to 2020, although the economic resilience of each megacity fluctuates to some extent, they are generally within a relatively stable range. According to the economic resilience, seven megacities can be divided into four levels, namely: Beijing, Shanghai, and Shenzhen have the highest economic resilience, Guangzhou and Tianjin have moderate economic resilience, Chengdu has a lower economic resilience, and Chongqing has the lowest economic resilience.

Secondly, from the perspective of development trends, compared to high and middle levels, megacities with lower economic resilience have shown a more significant upward trend, such as Chengdu and Chongqing. Only Shenzhen has shown an upward trend among the megacities with the highest economic resilience.

Thirdly, the highest and higher proportion of economic resilience in Chinese megacities depends as time passes, indicating that megacities’ economic resilience is weakening.

## Section 5: Discussion

Economic resilience provides a new insight for megacities to maintain sustainable economic growth under multiple shocks and risks. Accurately quantifying the economic resilience of megacities can provide data support for urban decision-makers to formulate policies. Therefore, the present study constructs an evaluation model of megacities’ economic resilience and verifies the model’s effectiveness using Chinese megacities as a case study. The present study compares and discusses the differences between the findings of the present study and previous studies, mainly in terms of evaluation indicators and evaluation results. The specific discussion is as follows:

The present study constructs the evaluation model of economic resilience from three dimensions: resistance, recoverability, and adaptability. Previous studies have mostly adopted these dimensions, such as the research of Martin & Sunley (2015), Huang et al. (2023), and Ma & Huang (2023). However, in terms of evaluation indicators setting (the initial selection of indicators comes from the theoretical and empirical research literature on economic resilience), the present study finds and eliminates three effect indicators (market potential, Industrial structure advancement, and new economic sectors development) through matrix calculation, and reintegrated the remaining indicators according to their respective levels. Four levels are divided, corresponding to the above three evaluation dimensions after layer merging. The first level has three indicators, the second level has seven, and the third level has five. The present study researches further analysis, screening, and classification of evaluation indicators that are relatively rare in previous studies.

The present study uses the constructed model to evaluate the economic resilience of Chinese megacities. Evaluation results show that Beijing, Shanghai, and Shenzhen have the highest economic resilience, Guangzhou and Tianjin have moderate economic resilience, Chengdu has a lower economic resilience, and Chongqing has the lowest economic resilience. It is consistent with the findings of Wang & Wei (2021), Wu et al. (2020) and Huang et al. (2023). For example, Wang & Wei (2021) proposed that among the 30 provinces in China, regions such as Beijing, Guangdong, and Shanghai had higher economic resilience [45]. Wu et al. (2020) found that there were significant spatial differences in economic resilience among 26 provinces in China [46]. Huang et al. (2023) found that the Pearl River Delta and the Yangtze River Delta have high economic resilience [16]. The comparison of the above verifies the effectiveness of the model constructed in the present study. By contrast, previous studies focused more on evaluating regional economic resilience, and the present study evaluates economic resilience at the urban level. Moreover, the present study identifies the distribution, differences, and trends in the economic resilience of Chinese megacities. The present studies contribute to the research scope and methodology.

## Section 6: Conclusion and policy implication

Accurately evaluating economic resilience can provide precise data support for urban decision-makers and is also a vital prerequisite for conducting dynamic research on economic resilience. There are currently no generally accepted methods for empirical evaluation or measuring economic resilience. With this in mind, the present study comprehensively utilizes methods such as DEMATEL, ISM, AHP, Entropy, and VIKOR to construct an evaluation model for the economic resilience of megacities and verifies the effectiveness of the model using Chinese megacities as a case study. The present study fills gaps and contributes to the research scope and methodology. The main conclusions of the present study are as follows:

Firstly, excluding three effect indicators, the evaluation model for the economic resilience of megacities includes a total of 15 indicators. These indicators can be divided into four levels, corresponding to three dimensions after layer merging: resistance, recoverability, and adaptability.

Secondly, using Chinese megacities as a case study to apply the model, the evaluation results found that Beijing, Shanghai, and Shenzhen have high economic resilience, Tianjin and Guangzhou have moderate economic resilience, Chengdu has low economic resilience, and Chongqing has the lowest economic resilience. In addition, megacities with lower levels of economic resilience exhibit a more significant upward trend, as well as the highest and higher proportion of economic resilience in Chinese megacities depending on time passes, indicating that megacities’ economic resilience is weakening. The evaluation results of the model are consistent with previous studies, and its effectiveness has been verified. However, the evaluation results obtained in the present study are more specific, precise, and focused on depicting the distribution differences and development trends of economic resilience at the urban level.

It is worth noting that although the present study has made every effort to enhance the rigor of research methods and processes, there are still some limitations. For example, due to the restriction of data, the evaluation model constructed in the presentation study is still not comprehensive enough to reflect the connotation and characteristics of economic resilience, and further completion is needed in the future. In addition, the present study uses seven megacities in China as examples to apply the model and verify its effectiveness with a relatively small sample size. In future research, the model can be validated using global megacities as samples to enhance the reliability of research conclusions.

Based on the above conclusions, some valuable policy implications for promoting a concerted improvement in the economic resilience of megacities are proposed. When formulating strategies to enhance the economic resilience of megacities, it is impossible to generalize, and localization and differentiation strategies should be implemented considering the actual situation of different cities. Urban decision-makers can use the model constructed in the present study to evaluate urban economic resilience and develop corresponding strategies for improving economic resilience. Specifically, for megacities with high economic resilience, such as Beijing, Shanghai, and Shenzhen, it is necessary to enhance their technological innovation capabilities while promoting the optimization and upgrading of industrial structures to diversify the risks that may arise from external shocks. For megacities with moderate economic resilience, such as Guangzhou and Tianjin, it is necessary to accelerate further the pace of industrial structure upgrading and fully utilize the advantages of economic development to achieve rapid improvement of urban economic resilience. For megacities with low economic resilience, such as Chengdu and Chongqing, they should continue to undertake industrial transfer from the eastern coastal areas, promote accelerated investment in urban infrastructure and supporting facilities, and enhance the resilience of the regional economy to external shocks.

## Abbreviations

AHP: Analytic Hierarchy Process
ANP: Analytic Network Process
BRIC: Baseline Resilience Indicators for Communities
CLES: Centre for Local Economic Strategies
DEMATEL: Decision Making Trial and Evaluation Laboratory
ISM: Interactive Structural Modeling
MOP: Multi-objective programming
PCA: Principal component analysis
RCI: Resilience Capacity Index
VIKOR: VIseKriterijumska Optimizacija i KOmpromisno Resenje
